# Sociocultural predictors of obligatory exercise in young men: A Polish-Chinese comparison

**DOI:** 10.3389/fpsyt.2023.1123864

**Published:** 2023-04-12

**Authors:** Shuai Guo, Bernadetta Izydorczyk, Małgorzata Lipowska, Sebastian Lizinczyk, Agata Kamionka, Urszula Sajewicz-Radtke, Bartosz M. Radtke, Taofeng Liu, Mariusz Lipowski

**Affiliations:** ^1^Faculty of Sport and Leisure, Guangdong Ocean University, Zhanjiang, China; ^2^Gdańsk University of Physical Education and Sport, Gdańsk, Poland; ^3^Institute of Psychology, Jagiellonian University, Krakow, Poland; ^4^Institute of Psychology, University of Gdańsk, Gdańsk, Poland; ^5^Central Board of Prison Service, Ministry of Justice, Warsaw, Poland; ^6^Laboratory of Psychological and Educational Tests, Gdańsk, Poland; ^7^School of Physical Education, Zhengzhou University, Zhengzhou, China; ^8^WSB University in Gdańsk, Gdańsk, Poland

**Keywords:** sociocultural, obligatory exercise, motivation for physical activity, body, mass media, cross-cultural

## Abstract

**Background:**

Obligatory exercise has been shown to have negative physical and mental effects on exercisers and is more prevalent among young people. However, there is limited research on obligatory exercise among young men. Social comparison theory offers a novel perspective to explore the relationship between sociocultural factors and obligatory exercise among young men, which offers an opportunity to understand potential factors contributing to obligatory exercise among young men across different cultures.

**Method:**

We recruited a purposive sample of young people aged 18-30 from Poland (n=79) and China (n=194). Participants completed self-report measures including the Sociocultural Attitudes Toward Appearance Questionnaire3, Inventory of Physical Activity Objectives, and Obligatory Exercise Questionnaire. In the data analysis stage, we examined the strength of the relationships between the independent variables and the dependent variable through multiple regression analysis, and tested the role of the mediating variables.

**Results:**

The main analyses revealed that Internalization-Athlete was a common direct predictor of obligatory exercise for both Polish and Chinese young men; that there were direct sociocultural predictors of obligatory exercise that were only used in relation to Polish or Chinese young men; and that social adaptation goals for motivation for physical activity mediated the development of obligatory exercise for Polish and Chinese young men, and that there were cross-cultural differences.

**Conclusion:**

Attention should be paid to their attitudes towards the idea of a muscular and athletic body and socially adapted physical activity motivations when understanding young men’s obligatory exercise, while also considering cross-cultural differences.

## 1. Introduction

Regular exercise positively affects the physical and mental health of young people, such as improving cardiovascular function, enhancing respiratory function, regulating mood, reducing depression and anxiety, and improving social adjustment (1–7). However, when the duration and frequency of exercise exceed safety upper limits, the adverse effects (skeletal muscle damage, metabolic disturbances, cardiovascular stress, etc.) may outweigh the positive effects (8–11). Researchers have studied this phenomenon using different terms such as exercise addiction (12), exercise dependence (13), compulsive exercise (14), and obligatory exercise (15). In this study, obligatory exercise was considered the most appropriate because it incorporates addiction, dependence, and compulsion. Obligatory exercise, defined by Polivy (16), is continued participation in physical activity despite the pain, lack of leisure time, interference with work or meaningful relationships, and the social consequences of indulgence. A systematic review on the prevalence of obligatory exercise risk confirmed that obligatory exercise risk in general exercisers is more prevalent in young people, including college students, probably due to body image (17). However, most of the available studies have focused on obligatory exercise in athletes, female college students, and adolescents (17–19), and few studies have focused on predictors of obligatory exercise in young men.

Social comparison theory suggests that people tend to compare themselves to others in terms of specific attributes (e.g., thinness, muscular) and that once the image promoted by the media is internalized, women and men will compare themselves to the media image and will act out for what they should look like and what they can do to achieve this goal (20). Internalization is the incorporation of specific values to the point that they become guiding principles or, as Thompson and Stice noted, “the extent to which an individual cognitively buys into” societal norms of size and appearance, to the point of modifying one’s behavior in an attempt to approximate these standards (21). Research with university student populations found that obligatory exercise was associated with sociocultural pressure to be thin, investment in appearance, weight problems, and body dissatisfaction (22, 23). Obligatory exercise among young people was associated with concerns about appearance, and the internalization of sociocultural standards of thinness would predict their obligatory exercise (22, 24, 25). Sociocultural influences are theorized to promote young people’s attitudes toward their bodies through three general processes: information, internalization of social standards, and responses to internalization (24, 26). Under the influence of globalization and industrialization, the esthetic standards of Asian, European, and American countries are merging and unifying with each other, and the ideal body shape of Western cultures is being widely promoted (27–29). However, cross-cultural differences in sociocultural attitudes about the body persist (30). In Europe, the Polish cultural identity is individualistic, seeking individuality and personal experience and emphasizing the autonomous individual’s respect toward the body norms of his or her approval; in Asia, the Chinese cultural identity is collectivistic, concerned with the playing of social roles and focusing on the individual’s physical submission to social norms (31–33). The literature review confirmed that obligatory exercise exists among young men in both Poland and China (17, 34, 35). Therefore, a cross-cultural comparison of young Polish and Chinese men brought up in different cultures could significantly improve the understanding of the predictors of obligatory exercise for young men in these cultures.

All human physical activities are driven and controlled by motivation (36). In addition to sociocultural factors, the motivational factors that support the development of obligatory exercise behavior are also worth mentioning. Recent literature has demonstrated that motivation appears to be an important factor in influencing physical activity and that young people’s attitudes toward physical and sporting activity will influence motivation and excessive physical activity intake (37). Pritchard and Beaver (15) showed that exercise motivation can predict obligatory exercise of young people, and the gender difference is noticeable, improved body tone, enjoyment, and perceived attractiveness predict the obligatory exercise of men, and improved body tone, fitness, and enhanced mood predict the obligatory exercise of women. A cross-sectional analysis of young people with an average age of 24.15 showed that their motivation for physical activity directly affects when, how often, and how they engage in physical activity (38). It is worth noting that research based on self-determination theory has demonstrated that extrinsic motivation for physical activity is associated with body dissatisfaction, internalization of sociocultural standards of body and appearance, and intrinsic motivation for physical activity is associated with higher body satisfaction and lower internalization of sociocultural standards of body and appearance (39–41). Karazsia and Crowther (42) demonstrated that Thompson’s three-factor model is applicable to explain body dissatisfaction and muscle development strategies in young men. Given these, this study not only measured the predictive role of sociocultural factors on obligatory exercise in young men but also assessed the mediating role of motivation for physical activity in the relationship between sociocultural factors and obligatory exercise in young men.

The literature sources suggest that the sociocultural factors proposed in this study as potential predictors of obligatory exercise have been analyzed in other studies and have typically focused on measures of sociocultural attitudes toward the body (22, 34). Given this, the independent variable in this study was sociocultural attitudes toward the body, which describes the extent to which sociocultural standards of body appearance are internalized (21). The dependent variable was obligatory exercise, describing attitudes and behaviors associated with exercise (43). The mediating variable was the motivation for physical activity, which describes how important a specific objective is to a person’s participation in physical activity (44). The physical activities here are performed without medical recommendations.

This article focuses on Polish and Chinese young men. The main aim of this study was to determine which sociocultural factors predicting obligatory exercise in young men are universal or common to each young man and which are specific to a particular cultural condition (Polish or Chinese culture). The authors believe that the stated research topic is not only related to obligatory exercise but can also become a health preference for a healthy and balanced physical activity treatment. The following research questions will be considered in this study.

1. Are there differences in the intensity levels of obligatory exercise, sociocultural attitudes toward the body, and motivation for physical activity between the young Chinese and Polish men surveyed?
2. Are there factors that together predict obligatory exercise in young Polish and Chinese men regarding sociocultural attitudes toward the body, and what are these factors?
3. What is the role of motivation for physical activity in the emergence of obligatory exercise among young Polish and Chinese men? Is it an intermediate variable in their development? If so, which factors in the research model mediated the development of obligatory exercise?

## 2. Materials and methods

### 2.1. Participants

The groups were chosen by purposeful sampling. Participants were recruited using convenience and purposive sampling methods. The inclusion criteria were: age (18–30 years old), Polish or Chinese nationality and growing up in that country (lived with the family from childhood to now in Poland or China for the respective groups), lack of physical disability or somatic diseases that prevents physical activity, and no requests for exercise have been received from doctors, students, or graduates in the humanities and social sciences.

The data used for this study were part of a large international research project registered in the Protocol Registration and Results System (ClinicalTrials.gov; https://clinicaltrials.gov/ct2/show/NCT04432038). In 2021, the project was conducted simultaneously in two academic cities in Poland (Krakow and Gdansk) and two academic cities in China (Beijing and Zhengzhou). The study authors trained qualified researchers (students and team members of the study authors) on research procedures and ethics, then conducted and coordinated the study simultaneously in China and Poland. Researchers disseminated information about the possibility of participating in the study among university students in the four cities and sent informed consent forms and questionnaires *via* email to volunteers who met the inclusion criteria. Volunteers who met the inclusion criteria were asked to help invite their classmates to participate in the study (non-random sampling method). Informed consent forms and questionnaires were sent to invitees *via* email by the researchers.

The study was planned to survey 150 young Polish men and 150 young Chinese men. However, a total of 125 young Poles and 257 young Chinese participated in the study. Forty-six young Poles and 63 young Chinese were excluded from the study due to errors in completing the questionnaire and failure to meet all inclusion criteria. The mean age of young Polish men was 24.3 (SD = 3.30), and the mean age of young Chinese men was 22.0 (SD = 2.61). The mean BMI for both young Polish and Chinese men is between the normal values of 20 and 25. The two groups of respondents were undergraduate and postgraduate students currently living in the surveyed Polish and Chinese cities and university graduates entering the workforce. Of all respondents, 72% were students, 25% were employed for wages, 83% were single and never married, 11% were in a Married or domestic partnership, and no respondents were widowed or separated. The respondents’ university majors were all in the humanities and social sciences; they had no experience as athletes and were not professional sports learners. All respondents reported that they participated in physical activity, and their average monthly participation was 14 (SD = 9.69).

### 2.2. Ethical approval

This study was conducted following national and international regulations and guidelines. The study was carried out in accordance with the Code of Ethics of the World Medical Association (Declaration of Helsinki) for research involving humans. The protocol of this study was approved by the Ethics Board for Research Projects at the Institute of Psychology, University of Gdansk, Poland (decision no. 33/2020).

### 2.3. Methods

The Sociocultural Attitudes Toward Appearance Questionnaire 3 (SATAQ 3) (21) was used to measure the variable sociocultural attitudes toward the body. The Inventory of Physical Activity Objectives (IPAO) (44) was used to measure the motivation for physical activity. The Obligatory Exercise Questionnaire (OEQ) (43) was used to measure the variable of obligatory exercise. In addition, demographic variables such as gender, nationality, age, height, and weight were collected. BMI is obtained by dividing the weight in kilograms by the square of the height in meters.

#### 2.3.1. The sociocultural attitudes toward appearance questionnaire 3

The Sociocultural Attitudes Toward Appearance Questionnaire 3 (SATAQ 3) by Thompson et al. (21), in Polish adaptation by Izydorczyk and Lizińczyk (45) and Chinese adaptation by Jackson and Chen (46), was used. The original version of the SATAQ 3 had 30 items and consisted of four subscales: Internalization-General (assesses the extent to which respondents internalization of sociocultural standards, consists of nine items, e.g., I compare my body to the bodies of TV and movie stars); Information (measures the frequency of seeking information about the sociocultural standards of body and appearance, consists of nine items, e.g., TV commercials are an important source of information about fashion and “being attractive.”); Pressures (assesses the level of pressure of sociocultural standards felt by a person, consists of seven items, e.g., I have felt pressure from TV or magazines to lose weight); and Internalization-Athlete (measures the level of internalization of athletic body shape, consists of five items, e.g., I wish I looked as athletic as sports stars). The respondents assessed each item of the SATAQ 3 by marking their answers on a five-point Likert scale. The Cronbach’s alpha coefficients for the four subscales in the presented study were as follows: Internalization-General (0.803 in Polish studies and 0.931 in Chinese studies), Information (0.766 in Polish studies and 0.922 in Chinese studies), Pressures (0.882 in Polish studies and 0.897 in Chinese studies), and Internalization-Athlete (0.746 in Polish studies and 0.839 in Chinese studies).

#### 2.3.2. The inventory of physical activity objectives

We used the Inventory of Physical Activity Objectives (IPAO) developed by Lipowski and Zaleski (44). The questionnaire consists of demographic variables, 12 physical activity objectives, and a motivational function of objectives scale. The first two parts of the questionnaire were investigated according to the needs of the study. Based on the research needs and drawing on Lipowski and Zaleski (44), and Sebire et al. (39) studies, this study divided the 12 physical activity goal items into three factors: physical development goals (containing four items; e.g., Physical fitness, being “in shape”), mental development goals (containing four items; e.g., Pleasure from physical activity), and social adjustment goals (containing four items; e.g., Company of other people). Respondents assessed the importance of the listed objectives by marking their answers on a five-point Likert scale, with 1 being not at all important and 5 being very important. The questionnaire obtained ideal Cronbach’s alpha coefficients in existing studies on both Chinese and Polish populations (44, 47). The Chinese version of the questionnaire was obtained using a standard forward-backward translation procedure. The Cronbach’s alpha coefficients were as follows: Polish version = 0.831, Chinese version = 0.855.

#### 2.3.3. The obligatory exercise questionnaire

We used the Obligatory Exercise Questionnaire (OEQ) by Thompson and Pasman (43). The questionnaire contains 20 items that measure attitudes and activities related to exercise (e.g., “Then I do not exercise, I feel guilty”). Respondents rated how often they experienced each exercise-related situation on a four-point Likert scale, with higher scores indicating a more substantial obligation to exercise. The questionnaire obtained ideal Cronbach’s alpha coefficients in existing studies on both Chinese and other national populations (34, 48). The Polish version and Chinese of the questionnaire were obtained using a standard forward-backward translation procedure. The Cronbach’s alpha coefficients for the OEQ were as follows: Polish version = 0.854, Chinese version = 0.869.

### 2.4. Statistical methods

The survey data were analyzed in Excel (Microsoft Office 365) and IBM SPSS Statistics 26. The steps in the statistical analysis were as follows.

Stage 1: Measure the mean, quartile, and standard deviation of all variables in the model, depending on the research objective and question.

Stage 2: Given that the tested variables were not all normally distributed, the Mann–Whitney U test was used to measure the significance of the difference between variables in the groups of Polish and Chinese.

Stage 3: The significance of differences in the strength of relationships and strength of correlations between variables in the study model was measured using Spearman’s rank correlation coefficient.

Stage 4: Measures the strength of the relationship between the independent and dependent variables using multiple regression analysis while testing the role of mediating variables. Six basic hypotheses of the multiple regression analysis were tested. The Shapiro–Wilk test significance values for the dependent variable (mandatory exercise) were 0.258 (China) and 0.385 (Poland), satisfying the condition of normal distribution, and other characteristics of the analyzed data allowed the use of multiple regression analysis. This stage aimed to find predictors of the dependent variable in the Polish and Chinese young male populations. Calculations were performed using the PROCESS macro for SPSS (49).

The study proposes an integrated model of hypothesized relationships between variables to explain the direct predictive role of sociocultural attitudinal factors about the body, the mediating role of motivation factors for physical activity in explaining the emergence of obligatory exercise. Model 4 in the PROCESS macro for SPSS was used to test for direct and indirect effects (49). The significance of the indirect effects was tested using bootstrapping, and a bootstrap sample of 5,000 was used to model the data distribution better. The confidence interval (CI) was 95%. The effect is insignificant if the confidence interval contains a zero value. Only unstandardized estimates could be calculated. The hypothesized relationships used for testing include only those variables for which there is a significant relationship.

## 3. Results

### 3.1. Sociocultural attitudes toward the body, motivation of physical activity, and obligatory exercise characteristics of young Polish and Chinese men (differences between the groups)

A comparative analysis of all variables in the research model between the Polish and Chinese groups shows significant differences between the two groups regarding certain variables (Table 1).

1. In terms of sociocultural attitudes toward the body, Polish and Chinese young males showed significant differences on variables other than Internalization-Athlete, and young Polish males showed significantly lower levels on average than Chinese males. This result suggests that young Polish and Chinese males show similar levels of recognition and acceptance of the athletic body ideals promoted by the mass media. However, young Chinese men show a higher level of acceptance of the general sociocultural standards of body and appearance promoted by the mass media, they seek information about sociocultural standards of body and appearance more frequently in the mass media, and they feel higher pressure from the sociocultural standards of body promoted by the mass media.
2. In terms of motivation for physical activity, young Polish and Chinese males do not differ significantly in terms of physical development goals, while significant differences are shown in terms of psychological development goals and social adaptation goals. Compared to young Chinese men, young Polish men are more focused on psychological development goals and less on social adjustment goals. This result suggests that young Polish men place more emphasis on motivation for physical activities such as pleasure, happiness, and stress elimination, while young Chinese men place more emphasis on motivation for physical activities such as adapting to social relationships and promoting socialization.
3. The comparative analysis of the quartiles also shows significant differences between Polish and Chinese young men in obligatory exercise. Young Polish men were significantly lower than young Chinese men. This suggests that Young Chinese males are more likely to undertake obligatory exercise.

**Table 1:** Comparative analysis of the Polish and Chinese young male groups according to the variables included in the research model.

Variables	Polish (*n* = 79)			Chinese (*n* = 194)			Difference
	Q1	Median	Q3	Q1	Median	Q3	*p*
Internalization-general	15.00	19.00	24.00	25.00	27.00	30.00	<0.001
Information	11.00	16.00	21.00	25.75	27.00	30.00	<0.001
Pressures	7.00	11.00	15.00	17.00	20.00	21.00	<0.001
Internalization-athlete	13.00	16.00	19.00	14.00	15.00	18.00	0.448
Physical	15.00	17.00	19.00	15.75	18.00	20.00	0.202
Psychological	15.00	18.00	20.00	14.00	17.00	19.00	0.019
Social	8.00	12.00	15.00	13.00	14.50	17.00	<0.001
Obligatory exercise	40.00	47.00	54.00	44.00	51.00	58.00	0.005

The significance threshold was set at 0.05; *n*, number of people; Q1, 25% Quartile; Q2, 75% Quartile.

### 3.2. The relation between studied variables among Polish and Chinese young men

From Table 2, it can be see that: among young Polish men, there were significant correlations between some of the variables in the study model. All factors of sociocultural attitudes toward the body except Pressures were significantly correlated with obligatory exercise, all factors of motivation for physical activity were significantly correlated with obligatory exercise, and all correlation coefficients were positive. Between sociocultural attitudes toward the body and motivation for physical activity, Internalization-Athlete and Physical development goals were significantly correlated, and the four factors of sociocultural attitudes toward the body were significantly correlated with social adaptation goals, and all correlation coefficients were positive.

**Table 2:** Correlation analysis for all variables for the groups of Polish and Chinese young men.

		Internalization-general	Information	Pressures	Internalization-athlete	Obligatory exercise
Physical	Polish	0.147	0.208	−0.010	0.273^*^	0.479^**^
	Chinese	0.041	0.062	−0.102	0.087	0.349^**^
Psychological	Polish	−0.059	0.040	−0.171	0.149	0.404^**^
	Chinese	−0.107	0.045	−0.095	0.010	0.300^**^
Social	Polish	0.379^**^	0.425^**^	0.314^**^	0.345^**^	0.466^**^
	Chinese	0.100	0.232^**^	0.150^*^	0.102	0.370^**^
Obligatory exercise	Polish	0.231^*^	0.226^*^	0.041	0.318^**^	1.000
	Chinese	0.165^*^	0.254^**^	0.188^**^	0.264^**^	1.000

^*^p < 0.05; ^**^p < 0.01.

Among young Chinese men, significant correlations were also found between some of the variables in the study model. Four factors on sociocultural attitudes toward the body were significantly and positively correlated with obligatory exercise, and three factors on motivation for physical activity were significantly and positively correlated with obligatory exercise. Between sociocultural attitudes about the body and motivation for physical activity, only Information and Pressures were significantly positively correlated with social adjustment goals.

To sum up, sociocultural attitudes about the body may predict compulsory exercise among young Polish and Chinese men but may only hold for some factors, and there may be cross-cultural differences. Some factors of motivation for physical activity may mediate between sociocultural attitudes toward the body and obligatory exercise, with cross-cultural differences. These results provide the premise for the next stage of model testing.

### 3.3. Predictors of obligatory exercise among young men in Poland and China

**Figure 1:**
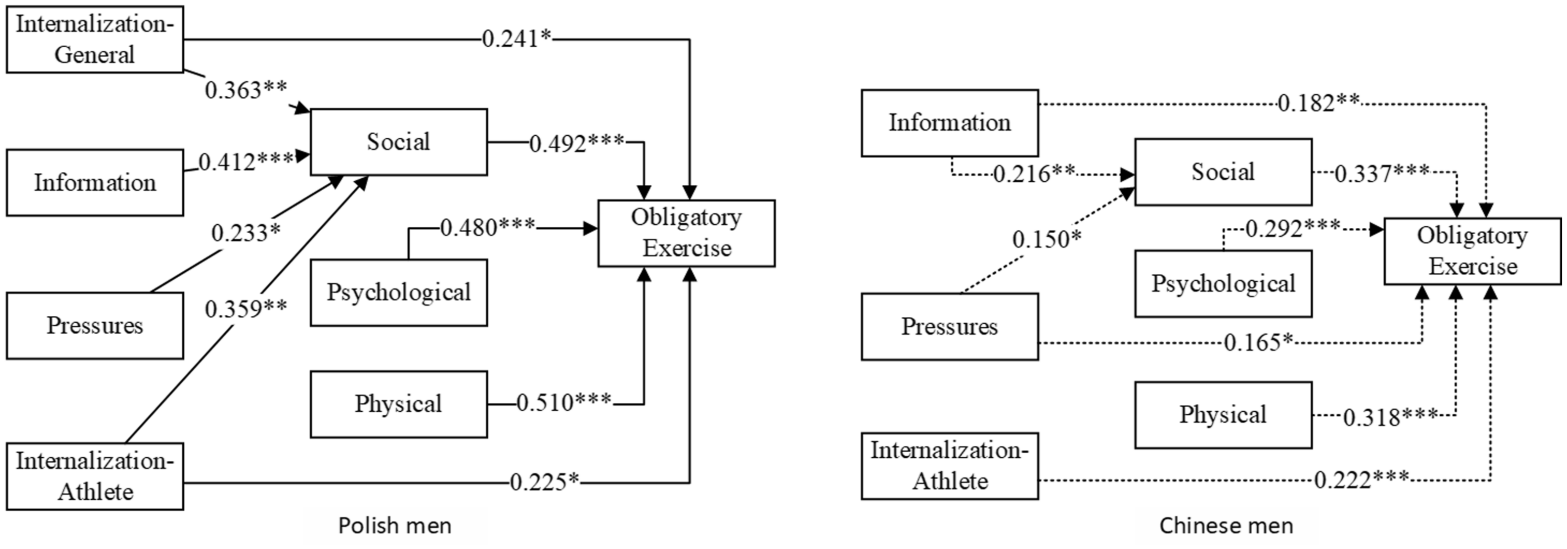
Sociocultural attitudes about the body (Internalization-General, Information, Pressures, and Internalization-Athlete) and motivation for physical activity (Physical, Psychological, and Social) as predictors of obligatory exercise among young Polish and Chinese men. Non-standardized estimates are presented; ^#^*p* < 0.05, ^**^p < 0.01, ^***^p < 0.001: to improve the readability of the presentation. Only paths with significance levels < 0.05 were retained.

Figure 1 illustrates the predictive role of the four factors regarding sociocultural attitudes toward the body among young Polish and Chinese men. In order to improve the readability of the presentation, only paths with a significance level < 0.05 were retained. Table 3 shows the model estimates for the three factors of motivation for physical activity as mediators of the four variables on sociocultural attitudes toward the body as predictors of obligatory exercise. The group effect is insignificant if a zero value is included between the Lower 95% CI and the Upper 95% CI.

**Table 3:** Model estimates of the four factors of sociocultural attitudes toward the body as direct predictors of obligatory exercise and indirect predictors through the three factors of motivation for physical activity.

		Estimates	SE	*p*	Lower 95% CI	Upper 95% CI
**Direct effects**						
Young Polish men	Internalization-General	0.241	0.099	0.017	0.044	0.438
	Internalization-Athlete	0.225	0.100	0.027	0.026	0.424
Young Chinese men	Information	0.182	0.068	0.008	0.048	0.316
	Pressures	0.165	0.068	0.017	0.030	0.300
	Internalization-Athlete	0.222	0.066	<0.001	0.092	0.352
**Indirect effects Social**						
Young Polish men	Internalization-General	0.179	0.056		0.078	0.295
	Information	0.210	0.067		0.100	0.360
	Pressures	0.125	0.060		0.012	0.248
	Internalization-Athlete	0.162	0.055		0.065	0.290
Young Chinese men	Information	0.073	0.027		0.026	0.131
	Pressures	0.052	0.024		0.009	0.103

Non-standardized estimates are presented.

From Figure 1, it can be seen that: among young Polish men, some of the pathways in the study model reached significant levels. Internalization-General and Internalization-Athlete were predictors of obligatory exercise. All three factors of motivation for physical activity were predictors of obligatory exercise. All four factors on sociocultural attitudes toward the body were predictive of social adaptation goals, while there was no predictive effect for the other two factors of motivation for physical activity.

Among young Chinese men, all factors on sociocultural attitudes toward the body, except Internalization-General, were predictors of obligatory exercise. All three factors of motivation for physical activity were predictors of obligatory exercise. Only Information and Pressures predicted the social adaptation goal of motivation for physical activity; the other factors of sociocultural attitudes toward the body did not have a predictive effect on motivation for physical activity.

Based on the results in Table 3, it can be seen that: the degree of mass media-driven internalization of the athletic body ideal is a common direct positive predictor of obligatory exercise among young males in Poland and China. The degree of internalization of universal sociocultural standards about the body promoted by mass media was only a direct positive predictor of obligatory exercise among young Polish men. The frequency of mass media messages seeking sociocultural standards about body and appearance and the level of pressure from mass media promotion of sociocultural standards about body and appearance was only direct positive predictors of obligatory exercise for young Chinese men.

Among young Chinese and Polish men, the frequency of mass media messages seeking sociocultural standards about body and appearance and the level of stress associated with mass media promotion of sociocultural standards of body and appearance indirectly predicted their obligatory exercise through the physical activity goal of adapting to social relationships and promoting socialization. This means that the social adaptation goal of physical activity is a common mediator of the development of obligatory exercise among young Polish and Chinese men. Furthermore, social adaptation goals for physical activity are also a mediator of young Polish men’s level of internalization of general sociocultural norms about the body promoted by the mass media and the level of internalization of the athletic body ideal as a predictor of obligatory exercise. However, this relationship was not present among young Chinese men.

## 4. Discussion

### 4.1. Similarities and differences among young Polish and Chinese men

The results of this study show that there are both similarities and significant differences between young Polish and Chinese men in terms of their sociocultural attitudes toward the body. Young Polish and Chinese men show similar levels of recognition and acceptance of the athletic body ideal as promoted by the mass media. However, compared to young Polish men, young Chinese men prefer to have the same appearance as figures in mass media such as TV or magazines, they seek information about body image and appearance more frequently from mass media, and they feel more pressure from mass media regarding appearance standards. Lipowska et al. (50) demonstrated in a comparative study of university students from Poland and Vietnam aged 19–25 that Young Polish men are significantly more satisfied with their physical appearance than young Vietnamese men due to cultural differences, young Polish men are concerned with their personalities and individual experiences, and they value their personal physical attractiveness; young Vietnamese men are susceptible to the social environment (significant social audience effect), and they value muscle, strength, and male-dominated social roles. Although there are no direct comparative studies of young Chinese and Polish people, Vietnam and China are part of the same East Asian cultural sphere and studies on them still provide support for this study. Furthermore, the study on sociocultural attitudes toward the body proved that young Chinese men show higher levels of Internalization-General, Information and Pressures (46, 51). Therefore, sociocultural attitudes toward the body differ significantly between young Polish and Chinese men, which could confirm the findings of other researchers.

In terms of motivation for physical activity, the results of this study show that young Polish and Chinese men show a similar level of emphasis on physical development goals; however, there are significant differences in the level of emphasis on psychological development and social adaptation goals. Compared to young Chinese men, young Polish men placed more importance on physical activity goals of pleasure, happiness, and stress elimination and less on physical activity goals of adapting to social relationships and promoting socialization. Established research on adults’ motivation for physical activity proves that the five most common motivations for adults (over 18 years old) to participate in physical activity are health and fitness, the improvement of physical appearance, enjoyment, socialization, and the psychological benefits it brings, and are influenced by gender, age, area of living, level of education, etc. (52–55). Wilczyńska et al. (47) demonstrated that culture moderates physical activity motivation between Poles and Chinese and that there are significant cross-cultural differences between the two in the company of other people, escape from everyday life, and managing stress. This provides support for the equally valued physical activity goals of young Polish and Chinese men in this study, as well as evidence that there may be cross-cultural differences in the physical activity goals of young Polish and Chinese men living in different regional and cultural environments. The study by Lipowska et al. (50) and Wilczyńska et al. (47) demonstrated that young Polish men brought up in a guilt-ridden culture focused on personal appearance and personal experiences; young Chinese men brought up in a culture of shame were vulnerable to the social environment. This supports the findings of this study that young Polish men place more emphasis on personal psychological development goals, while young Chinese men place more emphasis on social adjustment goals.

On the other hand, this study’s results also show significant differences in obligatory exercise between young Polish and Chinese men. Obligatory exercise was less likely among young Polish men than young Chinese men. Although there are no studies that directly show significant differences in obligatory exercise between Polish and Chinese young people. However, some studies have shown that solid sociocultural backgrounds influence people’s obligatory exercise behavior through different aesthetic standards (34, 56, 57). A systematic review by Reynolds et al. (58) demonstrated that obligatory exercise was associated with multiple sociocultural factors (e.g., family, peers, and media) in young people and that social comparison, body-related information, and pressure to conform to body ideals predicted this relationship. The differences in obligatory exercise in this study also provide the premise for the differential predictors of young Polish and Chinese men raised in different cultures, providing the basis for the second question of the study.

### 4.2. Predictors of obligatory exercise in the study group of young Polish and Chinese men

#### 4.2.1. The direct predictive role of sociocultural attitudes toward the body in obligatory exercise

This study aimed to determine which sociocultural factors predicting obligatory exercise in young men are common or universal for each young man and which are specific to a particular cultural condition (Polish culture or Chinese culture). The findings suggest that Internalization-Athlete is a common direct positive predictor of obligatory exercise for young men in Poland and China, Internalization-General is only a direct positive predictor of obligatory exercise for young men in Poland, and Information and Pressures are the only direct positive predictor of obligatory exercise for young men in China. Importantly, this study suggests that young men’s acceptance of the athletic body ideal promoted by the mass media will positively influence their obligatory exercise. This result supports other authors’ evidence across countries and regions that young men place greater emphasis on muscle and strength, which may lead them to engage in obligatory exercise (12, 22, 59–64). The study also suggests that young Polish men’s recognition of universal sociocultural norms about the body as promoted by mass media is a direct positive predictor of their compulsory exercise. The frequency with which young Chinese men seek information about sociocultural standards of the body promoted by mass media and the level of perceived pressure from body and appearance sociocultural standards promoted by mass media would directly and positively predict their obligatory exercise. According to Lipowska et al. (50), young Polish men focus on individuality and personal body experiences, while young Chinese men are more concerned with personal social roles. Thus, cultural background idiosyncrasies may lead young Polish men to value active personal recognition and acceptance of social culture, whereas young Chinese men value passive personal adaptation and fit into social culture. The study by Izydorczyk et al. (65) also confirmed that the higher the recognition and acceptance of general sociocultural attitudes about the body promoted by the mass media among young Polish men, the more likely they were to exhibit bulimic behavior. According to social comparison theory (20), young Polish men may exercise more frequently and with greater intensity to maintain their athletic body shape and attractiveness to counteract the adverse effects of overeating. On the other hand, to maintain their male-dominated social role, young Chinese men’s heightened attention to mass media information about sociocultural standards of the body and perceived higher pressure about sociocultural standards of the body may translate into higher frequency and intensity of exercise. Fan et al. (34) also confirmed that Information and Pressures were highly associated with obligatory exercise among young Chinese men. These support the findings of this study to some extent.

#### 4.2.2. Motivation for physical activity as a mediator between sociocultural attitudes toward the body and obligatory exercise

The results of this study also suggest that motivation for physical activity is a predictor of obligatory exercise in both Polish and Chinese young men, that motivation for physical activity mediates the relationship between sociocultural attitudes toward the body and obligatory exercise, and that there are both general mediators that apply to both young Polish and Chinese men, and mediators that apply only to a specific culture (Polish or Chinese culture). All three factors of motivation for physical activity predict obligatory exercise in Polish and Chinese young men. However, only social adaptation goals mediated the Information and Pressures factors in predicting obligatory exercise in young Chinese and Polish men. In addition, social adaptation goals mediated the Internalization-General and Internalization-Athlete in predicting obligatory exercise in young Polish men. This suggests that the frequency with which young men seek out information about sociocultural standards of the body promoted by the mass media and the level of perceived stress about sociocultural standards of the body promoted by the mass media indirectly positively predict their obligatory exercise by adapting to social relationships and promoting socialized physical activity goals. Studies on social identity theory confirm that higher levels of attention to information and perceived stress about sociocultural standards of the body promoted by the mass media among young people indicate greater concern about the socialization of one’s body and social identity, and lower levels of current body satisfaction (66–68). According to social comparison theory, this phenomenon may translate into specific goals and goal-directed behavior in young people (69). The study by Pritchard and Beaver (15) confirmed that motivation for physical activity in young men in tone, enjoyment, and attractiveness was a predictor of their obligatory exercise. This study also shows that the acceptance of universal sociocultural standards about the body promoted by the mass media and the receptivity to the athletic body idea promoted by the mass media among young Polish men indirectly positively predicted their obligatory exercise by adapting to social relationships and promoting socialized physical activity goals. However, this relationship does not exist among young Chinese men. Studies by Malchrowicz-Mośko et al. (57) and Lopuszanska-Dawid et al. (70) confirm that under the influence of media and interpersonal interactions, young Polish men internalize body images promoted by the media as their ideal body shape, which may lead them to engage in specific physical activities in order to enhance their physical attractiveness and social identity. However, there are few relevant studies on young Chinese men, which makes the present study significant. Furthermore, a systematic review by Teixeira et al. (71) indicated that motivation was a key factor in supporting sustained exercise and that more autonomous forms of motivation, such as pleasure and personal achievement, were positively associated with exercise in young people and predicted the duration and frequency of their exercise. The results of this study further support these studies.

The results of the resulting analysis suggest that prevention interventions for obligatory exercise in young men should pay attention to the promotion of a reasonable athletic body ideal and to the assessment of motivation for physical activity and specific sociocultural contexts. The authors recommend the inclusion of such interventions in health universities and national fitness programs. The practical implications of the authors’ study revolve around the professionals involved in physical activity and health programs for young people. Measures of sociocultural attitudes toward the body and motivation for physical activity may be necessary in the practice of educators, physicians, psychologists, and other specialists who support the healthy development of young men.

### 4.3. Limitations of the study

This study is a rare, even unique, opportunity to make cultural comparisons between two different people. However, this study also has noteworthy limitations. Firstly, while sufficient for statistical analysis, the study’s sample size is not large. Secondly, the research analysis provides an opportunity to compare two distinctly different cultures, which has been lacking in previous literature. Thirdly, although the study focused on compulsive motor cognition rather than motor behavior, the lack of a measure of motor behavior may be a limitation. Future research will consider combining motor behavior with compulsive motor cognition and work toward larger sample sizes and different age groups.

## 5. Conclusion

The cultural differences between young Polish and Chinese men indicate that young Chinese men show higher intensity on the independent variables Internalization-General, Information, and Pressures. However, Chinese and Polish young men showed similar strengths on the independent variable Internalization-Athlete. Young Chinese men showed higher intensity on the social adaptation goal of motivation for physical activity and lower intensity on the psychological development goal of motivation for physical activity than young Polish men. They were more likely to engage in obligatory exercise.

The findings suggest that the sociocultural factor of Internalization-Athlete is a common direct predictor of obligatory exercise for both Polish and Chinese young men, Internalization-General is a direct positive predictor of obligatory exercise for Polish young men only, and Information and Pressures were the direct positive predictors of obligatory exercise for young Chinese men only. Motivation for physical activity positively predicted obligatory exercise among young Polish and Chinese men.

It is worth mentioning that the social adaptation goal of motivation for physical activity mediates the prediction of obligatory exercise by sociocultural attitudes toward the body. The sociocultural factors Information and Pressures indirectly and positively predicted obligatory exercise in young Polish and Chinese men through the social adaptation goals of motivation for physical activity. The sociocultural factors Internalization-General and Internalization-Athlete indirectly and positively predicted obligatory exercise in young Polish men through the social adaptation goal of motivation for physical activity. However, BMI does not act as a mediator for the development of obligatory exercise in young men.

## Data availability statement

The raw data supporting the conclusions of this article will be made available by the authors, without undue reservation.

## Ethics statement

The studies involving human participants were reviewed and approved by the Ethics Board for Research Projects at the Institute of Psychology, University of Gdansk, Poland (decision no. 33/2020). The patients/participants provided their written informed consent to participate in this study.

## Author contributions

BI, MałL, and SG contributed the conception and design of the study. SG was primarily responsible for the manuscript writing and performed initial data analysis. SL performed a statistical analysis check. BI, MałL, SL, and AK commented on the manuscript revisions. MałL, MarL, BI, US-R, BR, and TL were responsible for data collection. MarL, BI, and MałL were responsible study supervision. All authors contributed to the article and approved the submitted version.

## Funding

MarL has been supported by the Polish National Agency for Academic Exchange under the Urgency Grants program (BPN/GIN/2021/1/00010/U/00001).

## Conflict of interest

The authors declare that the research was conducted without any commercial or financial relationships that could be construed as a potential conflict of interest.

## Publisher’s note

All claims expressed in this article are solely those of the authors and do not necessarily represent those of their affiliated organizations, or those of the publisher, the editors and the reviewers. Any product that may be evaluated in this article, or claim that may be made by its manufacturer, is not guaranteed or endorsed by the publisher.
